# Supplementary material for: A supertough electro-tendon based on spider silk composites
Source: Nat Commun. 2020 Mar 12;11:1332. doi: 10.1038/s41467-020-14988-5 (PMC7067870; doi:10.1038/s41467-020-14988-5)
Supplement: Supplementary file 1 — Supplementary Information [file 41467_2020_14988_MOESM1_ESM.pdf]

Supplementary information for:

A supertough electro-tendon based on spider silk composites

Liang Pan[1] et al

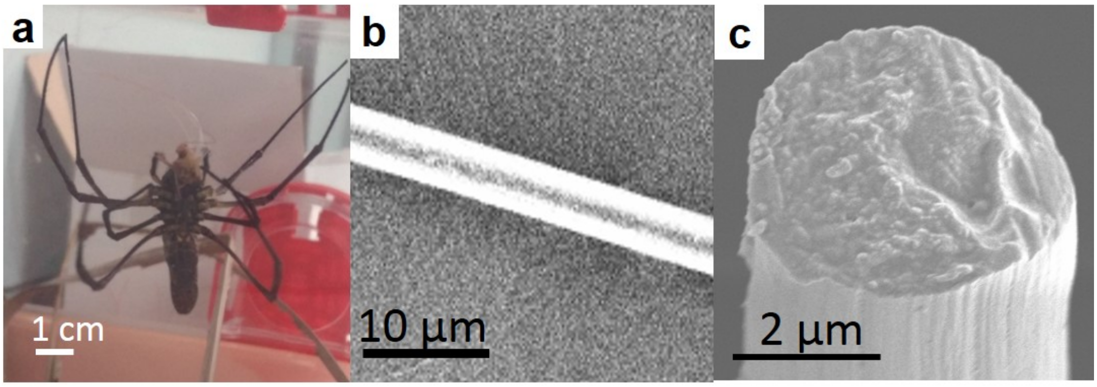

Supplementary Figure 1 │Spider silk production and characterization. (a) Optical photograph showing the spider, *Nephila pilipes,* feeding on a locust in an 80 cm×60 cm×40 cm vivarium. The spider was kept at humidity above 65% and temperature about 25°C. The spider was fed live locusts and flies three times a week. We collected the spider silk every two weeks using a scalpel and transferred the silk onto a rigid frame. (b)(c) SEM images show the dragline silk has a diameter of 3~4 μm, and has a smooth surface.

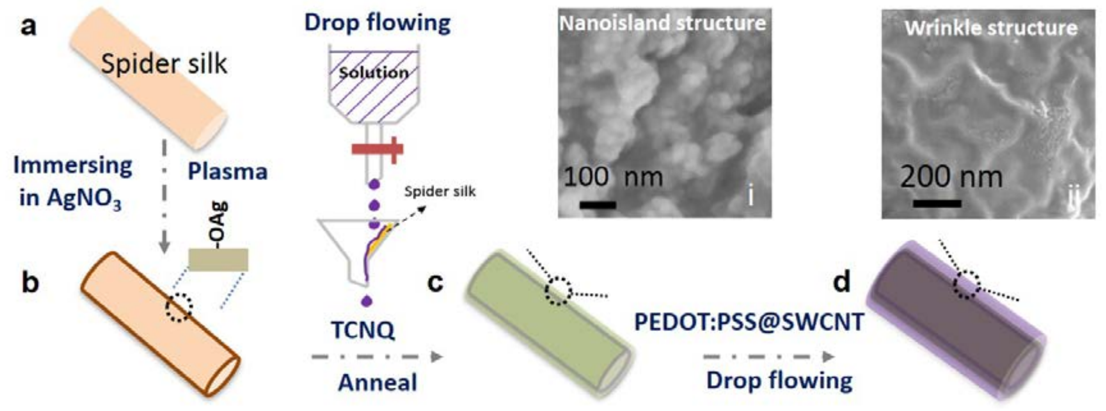

Supplementary Figure 2│Fabrication of the spider silk composite. (a) The collected spider silk (mainly dragline silk) was rinsed 3 times using ethanol (Absolute, 99.9%) and dried at 80°C for about 12 hours. Then, the silk was hydrophilized by plasma treatment at O_2 atmosphere for 10 min. (b) The hydrophilized silk was immersed in 0.1 mol/L ethanolic $AgNO_3$ for 30 min. (c) We coated with 0.01 mol/L TCNQ of ethanol by the method of drop flowing (shown in Supplementary Fig. 2 and Fig. 2-i). A lay of nano-island structure was formed after annealing at 100°C for 5 min. (d) The modified spider silk was coated with PEDOT:PSS@SWCNT using the drop flowing method. Wrinkles form on the coating because of the intrinsic shrinkage of spider silk induced by water in PEDOT:PSS. Insets i and ii show SEM images of the nano-island (i) and wrinkled structures of the coating layer (ii).

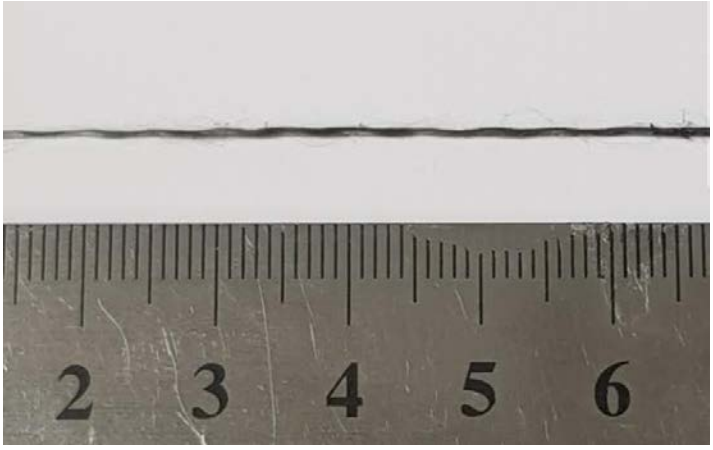

Supplementary Figure 3 │**Optical image of modified spider silk.** The color of the spider silk changed into black after coating with PEDOT:PSS@SWCNT.

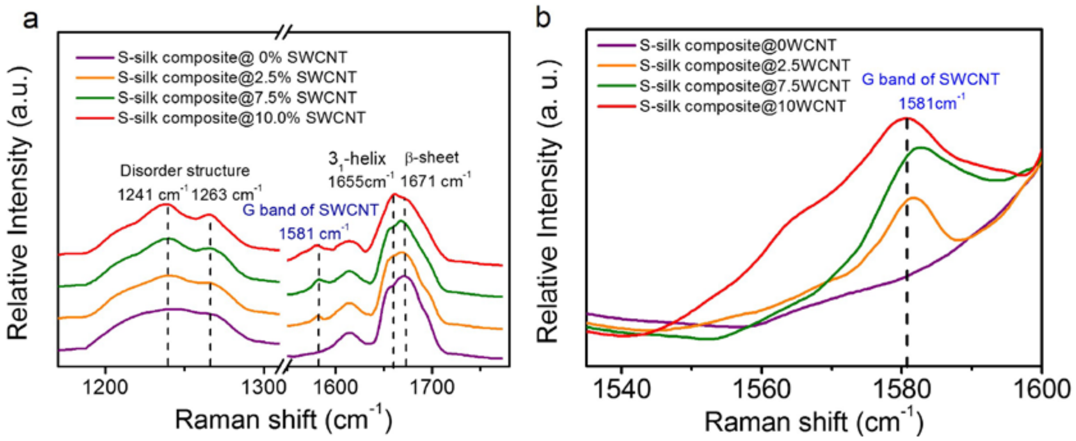

Supplementary Figure 4 │Raman spectrum of spider silk containing different wt% SWCNT. The samples of the cross-section (Fig.1d) were obtained by cutting the encapsulation epoxy resins with spider silk along the silk direction using a sharp blade, after freezing in liquid nitrogen. (a) The peaks at 1,241cm^{-1}, 1,263cm^{-1} are attributed to the disordered structure while peaks1,655cm^{-1} and 1,671cm^{-1} are attributed to 3_1-helix and β-sheet in spider silk [1]. The peak at 1,581cm^{-1} is ascribed to the G band of SWCNT. The D band of SWCNT at 1,250cm^{-1} was too weak to detect in our case. (b) Zoomed-in spectrum shows the intensity of the peak at 1581cm^{-1} increased gradually with wt% of SWCNT.

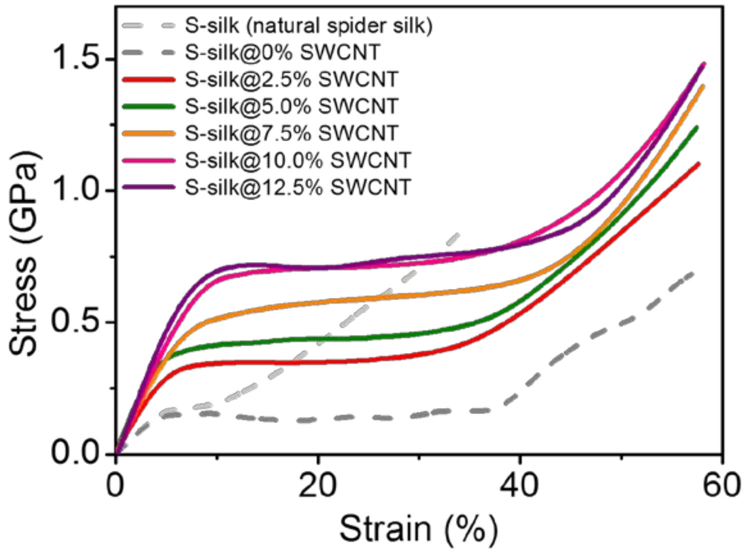

Supplementary Figure 5 │Stress-strain curves of different S-silk composites compared to natural S-silk. S-silk (natural spider silk), S-silk@0%SWCNT and S-silk with different wt% of SWCNT. S-silk coated with SWCNT showed enhanced toughness, Young's modules and strength until 10 wt% SWCNT. Beyond 10 wt% SWCNT, the stress-strain curve did not change due to poor dispersion of SWCNT in water.

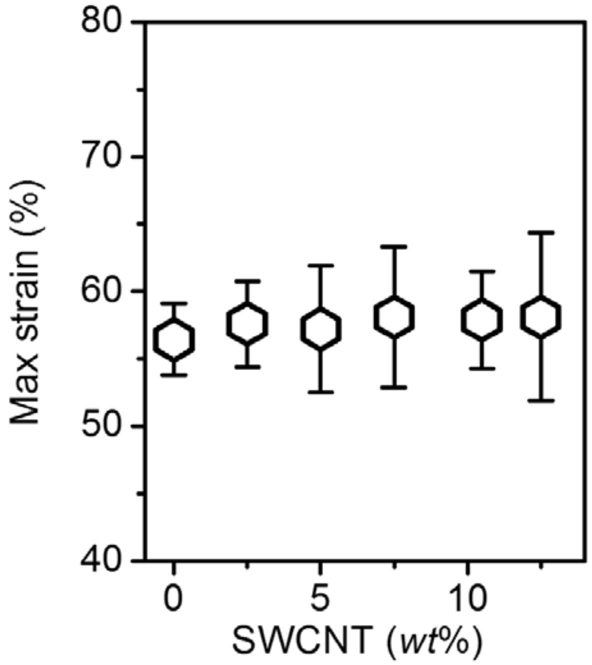

Supplementary Figure 6 │ Max strain changed with increasing wt% of SWCNT. With increasing of SWCNT content, the maximum strain of S-silk composite was ~60%. The error bar shows standard deviations based on 50 independent samples.

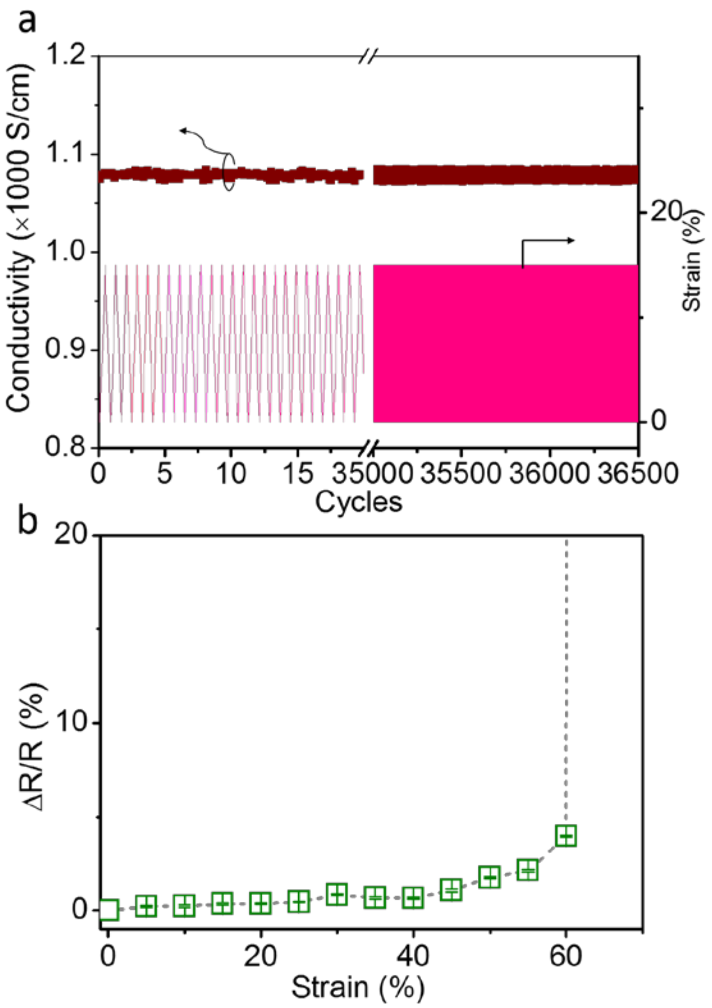

Supplementary Figure 7│Electrical properties of S-silk composite along with strain. (a) Graph shows the conductivity of the S-silk@10 wt% SWCNT remained at 1,070 S/cm even after 36,5000 cycles of 0 to 15% strain. Here, we chose 15% as reference because this is the max strain a human tendon can withstand. (b) Graph shows the rate of change of resistance of S-silk composite@10% SWCNT under strain from 0% to breakage. The ratio of ΔR/R was about 5% even at 60% strain. This low change in resistance is due to the wrinkled structure of the conductive layer, formed after intrinsic shrinkage of S-silk in aqueous solution. The error bar shows standard deviations based on 50 independent samples.

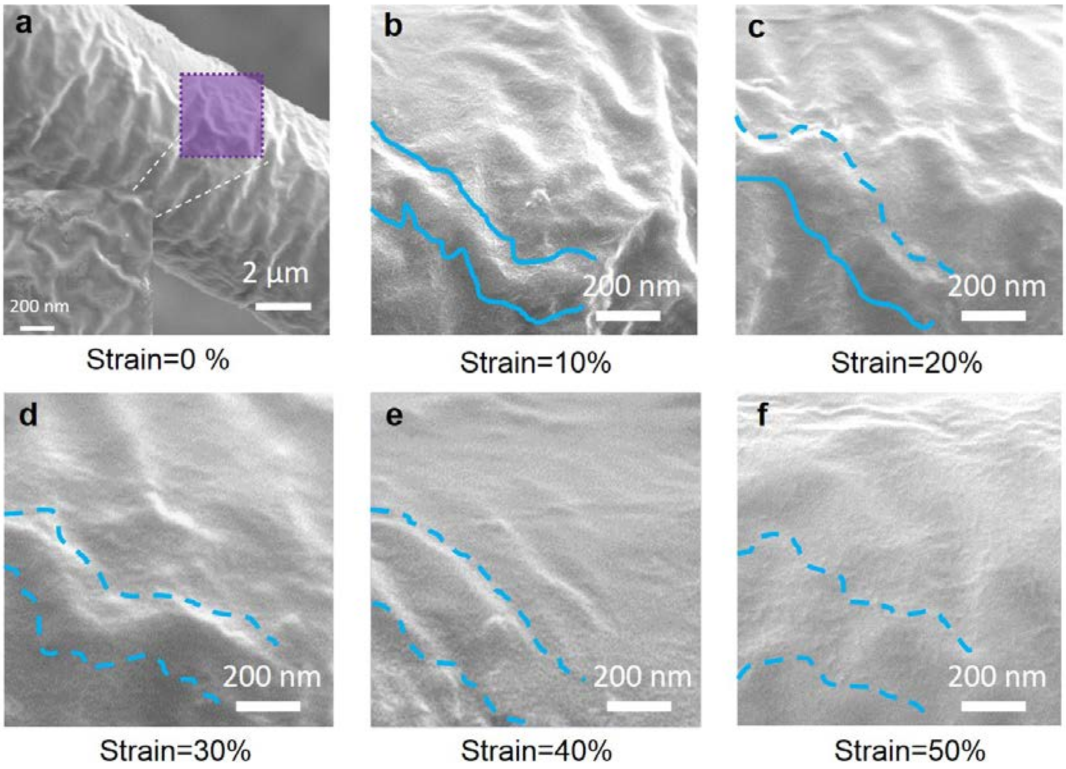

Supplementary Figure 8 │Change in morphology of S-silk composites with increasing strain. SEM images show that when strained, wrinkled areas (blue lines) on the conductive layer of the S-silk composites gradually flatten, preventing any changes in the conductive path. This allows the conductivity of the S-silk composites to remain unchanged even after > 1,000 cycles of stretching and compression between 0 to 15% strain. No cracks were seen during stretching. The wrinkled structures recovered when strain was below 20%. For strains above 20%, the wrinkle area will appear at other regions when stretched and compressed.

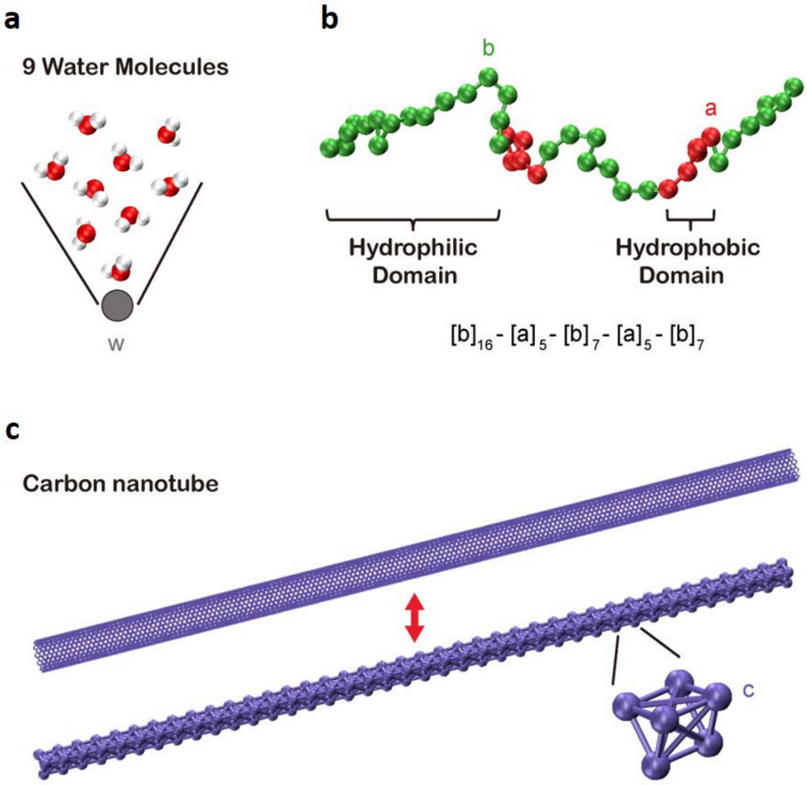

Supplementary Figure 9 | Models of DPD simulation. DPD models for (a) water molecule, (b) silk peptide and (c) carbon nanotube. Every 9 water molecules are represented by one hydrophilic "w" bead. Silk peptides are described as multiblock copolymer chains composed of hydrophobic "a" and hydrophilic "b" beads, with each bead representing 3 amino acids in the β-sheet crystalline and amorphous domains, respectively. (B) DPD model of a single silk peptide described in this study is represented by $[b]_{16} - [a]_5 - [b]_7 - [a]_5 - [b]_7$ sequence as described previously. The coarse-graining (CG) beads are connected by harmonic potentials. To include the single-wall carbon nanotube (SWCNT) into the DPD simulations, each SWCNT is also described as CG beads connected by harmonic potential, as shown in (c). To maintain the structure of the SWCNT, the cross section of the SWCNT is represented by three beads, as shown in (c).

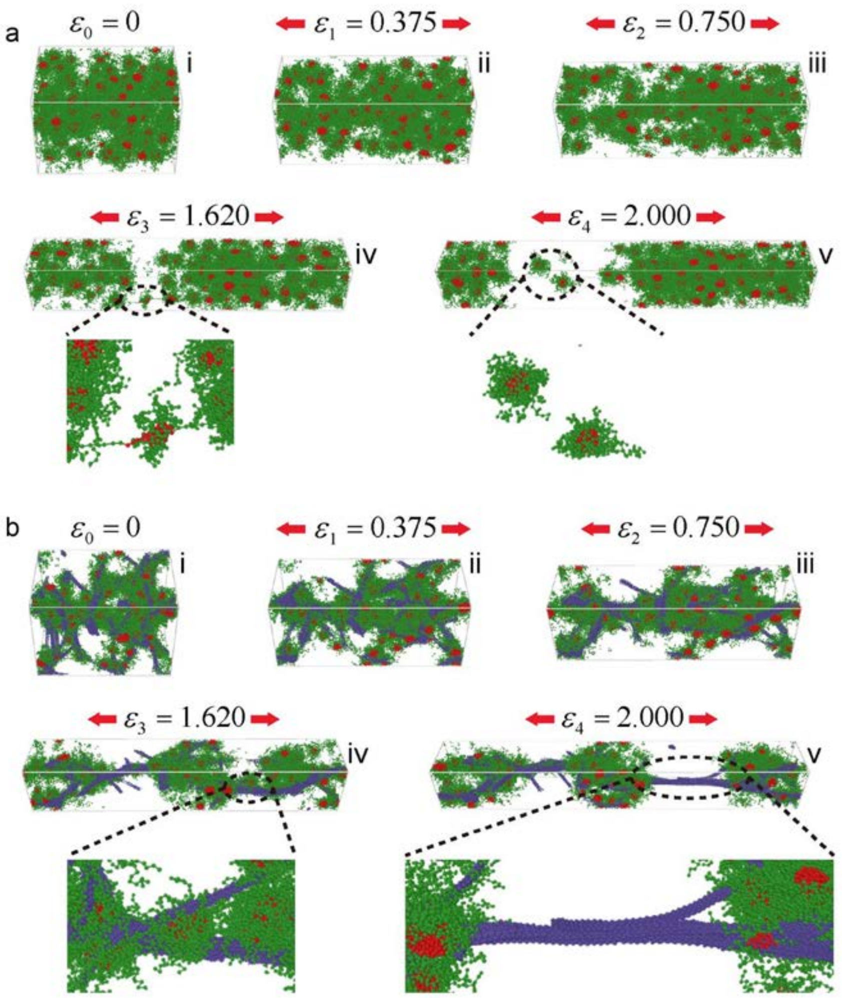

Supplementary Figure 10 │Dynamic behavior of the S-silk composite at microscopic scale under different strain based on DPD simulation. At the beginning of applying strain from 0 to 0.75 (here strain is normalized strain), the amorphous regions in natural S-silk gradually unfolded (a). In the case of the S-silk composite (b), the hydrophobic interaction between SWCNTs and silk proteins induced high strength. With increasing strain, the composite experiences higher normalized stress due to the bridge effect between SWCNTs and the silk proteins. The S-silk fractured more easily than S-silk composite at a lower critical strain, showing the composite is much tougher. In the simulation, we defined fracture as the number of bridges crossing any cross section to be less than 1. Red: crystalline structure, beta-sheet structure; green: amorphous structure; purple: SWCNT.

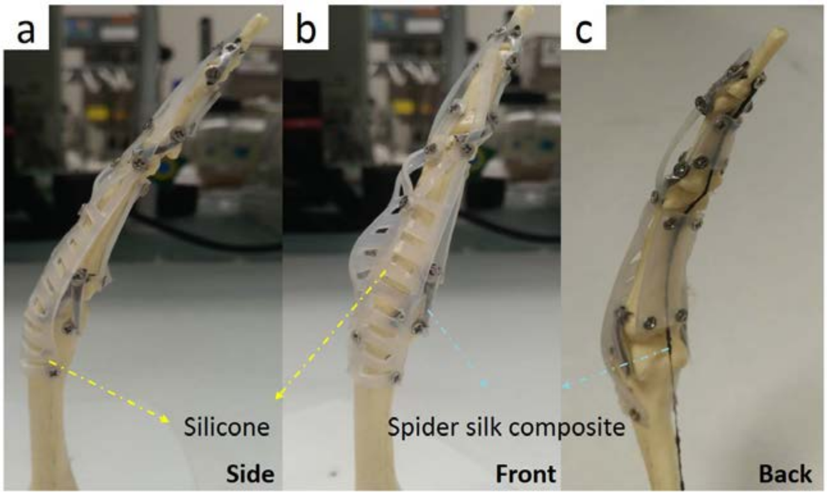

Supplementary Figure 11 │The humanoid robotic finger described in this study. The robotic finger was 3D-printed to the same size of a human finger bone. Silicone was used as the extensor hood while our S-silk composite acted as tendons and ligaments. There are no hinges in our humanoid finger. Screw spike was used to fix every part of the finger. S-silk changed from white to black after coating with PEDOT:PSS@SWCNT. (a), (b) and (c) show the side, front and back view of the finger, respectively.

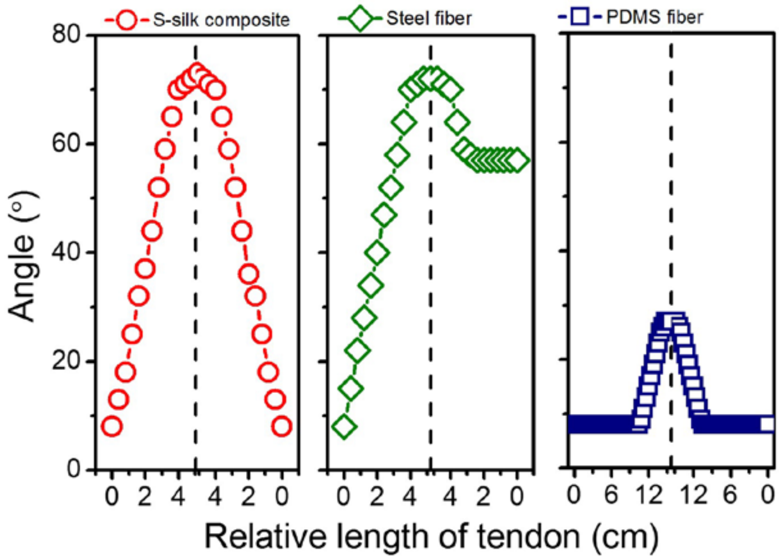

Supplementary Figure 12 | Bending action of robotic finger based on S-silk composite, steel fiber and PDMS fiber. (a-c) Fingers containing S-silk composite could bend fully (with angles changing from 8° to 73°) whereas those with steel and PDMS fibers could not due to poor toughness.

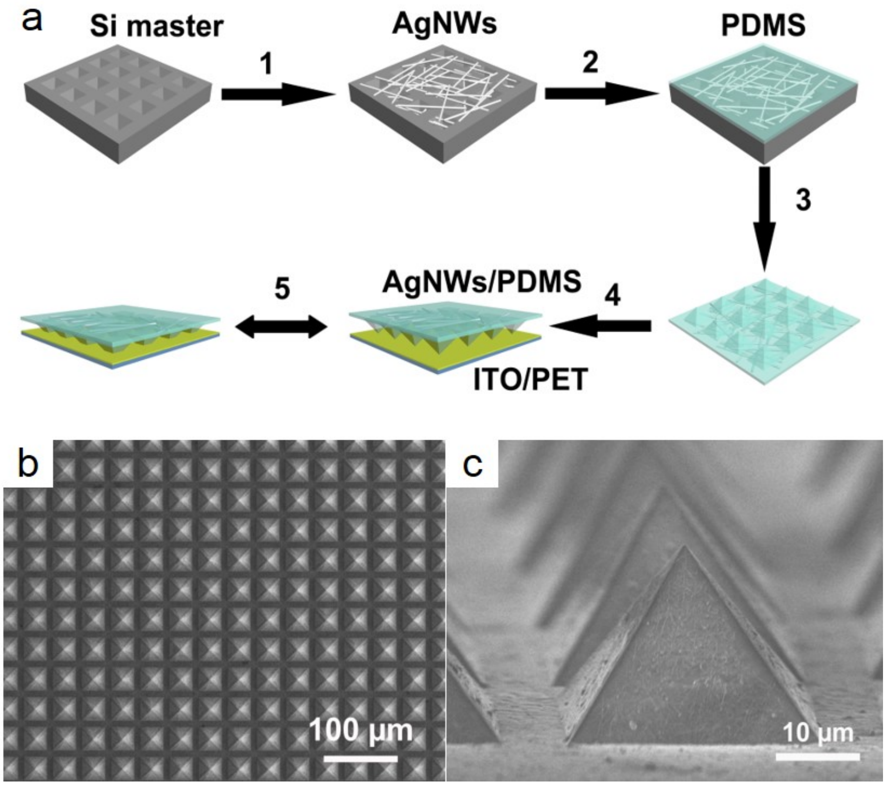

Supplementary Figure 13 │Fabrication of the pressure sensor. (a) Schematic showing the fabrication process for the pressure sensor. (b) (c) SEM images showing the pyramidal structures of the pressure sensor at different scales. The sensitivity is about 24.8 kPa^{-1}. This sensor can detect pressures from 0-1kPa in < 4 ms, which is enough for our grasping experiments. We assembled the pressure sensor on the tip of the index finger and connected it to the electro-tendon with a reference resistor of 100 kΩ.

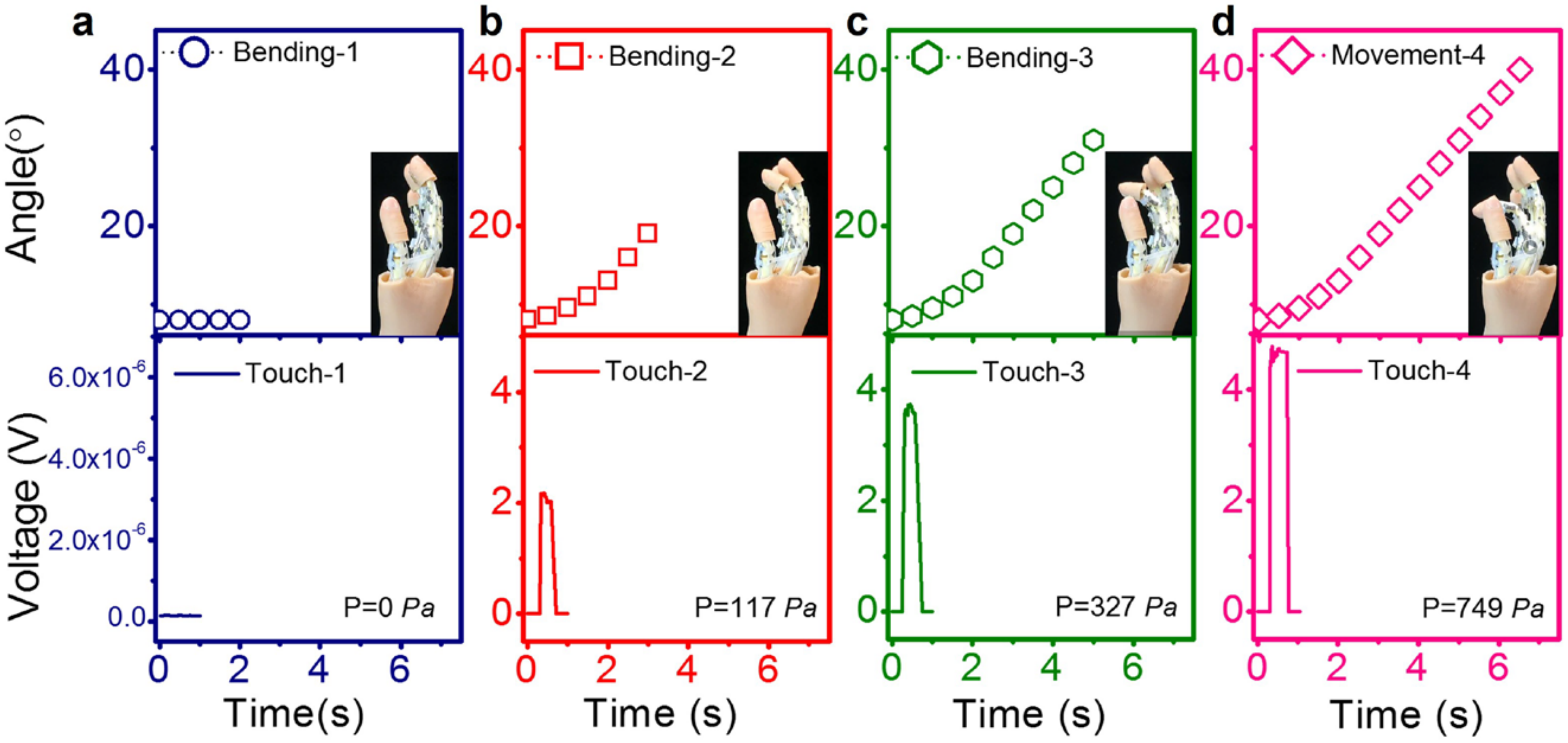

Supplementary Figure 14 │Bending process of the humanoid robotic hand. Top panels of (a) (b) (c) (d) show the humanoid robotic hands bending in response to different pressures (0, 117, 327 and 749 Pa). Bottom panels show the voltage signals produced when we touched the pressure sensor. Higher forces resulted in greater bending angles.

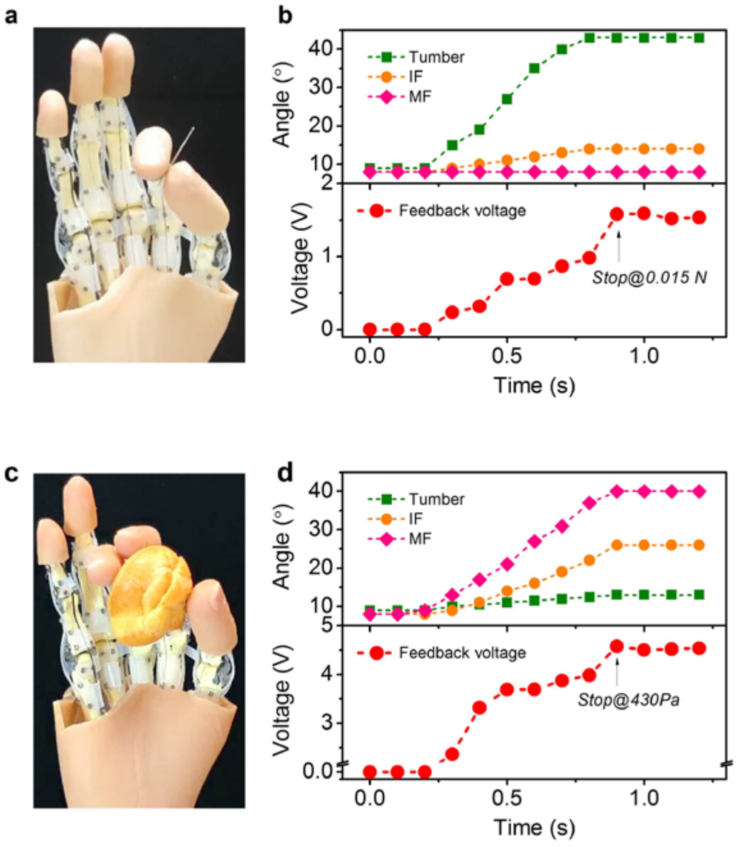

Supplementary Figure 15 | The processes of grasping a needle and a puff. In the case of the needle, the hand stopped moving when the force detected by the pressure sensor was over 0.012 N (in this case, the force recorded was 0.015 N). Because the touch area between the needle and the sensor cannot be defined, here, we used the force detected by the sensor when the hand grasped the needle as reference. In the case of the puff, the hand was programmed to stop moving when the pressure is above 400 Pa (in this experiment, the pressure recorded was 430 Pa). This robotic hand based on the electro-tendon is clearly dexterous enough to perform basic grasping functions that are useful for day-to-day activities.

Every activity of our robotic hand such as grasping things needs a lot of training processes. We take the process of grasping balloon as example. Firstly, we manual stop the hands without pressure sensor when it grasped the balloon. During these training processes, we figured out the appropriate bending speed of every finger such as thumb, index finger and middle finger when the robotic hand harmoniously grasped the balloon. After we achieved the bending speed, we assembled the pressure sensor on the finger to measure the pressure. Then, we used the pressure as a

reference to programmed control, instead of manual control, the stopping of the hands when it harmoniously grasped the balloon. In the case of manuscript, the pressure of index finger was ≥ 170Pa. Thus, we don’t need assemble the pressure sensor on every finger.

Supplementary Table 1 | Different composite materials and their conductivity and toughness values compared to the S-silk composite reported in this work.

	Composite	Conductivity (S/cm)	Toughness (MJ/m^3)*	Reference
**1**	RGG-Ag NWs	800	~16	Z. Xu, et al. *Adv. Mater*. **25**, 3249-3253 (2013).
**2**	Carbon fiber**	6,000	~30	S. Chand, et al. *J. Mater. Sc*. **35**, 1303-1313 (2000).
**3**	EGaIn-SEBS	34,000	~55	S. Zhu *et al.*, *Adv. Funct. Mater*. **23**, 2308-2314 (2013).
**4**	PANI-CNT	100	~40	J. J. Vilatela, et al. *Chem. Mater*. **27**, 6901-6917 (2015).
**5**	MWNT-PU	10	~70	M. K. Shin *et al.*, *Adv. Mater*. **22**, 2663-2667 (2010).
**6**	SIBS:P3HT	0.38	~85	A. J. Granero *et al.*, *Adv. Funct. Mater*. **21**, 955-962 (2011).
**7**	AgNWs/PDMS	5,285	~1	F. Xu, et al. *Adv. Mater*. **24**, 5117-5122 (2012).
**8**	Ag(nanoparticles)/SBS	2,200	0.38 (<1)	M. Park et al., *Nat. Nanotech.* 7, 803-809 (2012).
**9**	MWNTs-PDMS	100	~3	Y. Y. Huang, et al. *Adv. Funct. Mater*. **20**, 4062-4068 (2010).
**10**	PEDOT:PSS/PDMS	70	~2	J. Y. Oh, et al. *Adv. Mater*. **28**, 4455 (2016).
**11**	SWNCT/PDMS	100	~1	T. Sekitani *et al.*, *Nat. Mater*. **8**, 494-499 (2009)
**12**	CNTs/PVA	115	~7	W. X. Cao *et al.*, *Adv. Funct. Mater*. **27**, 1701061 (2017).
**13**	Sliver	621,000	~1.2	*https://www.tibtech.com/conductivite.php?lang=en_US* R. L. Templin, et al. *J. Aeronaut. Sci*. **7**, 189-198 (1940).
**14**	Copper	585,000	~1.5	*https://www.tibtech.com/conductivite.php?lang=en_US* R. L. Templin, et al. *J. Aeronaut. Sci*. **7**, 189-198 (1940).
**15**	Gold	442,000	<1	*https://www.tibtech.com/conductivite.php?lang=en_US* R. L. Templin, et al. *J. Aeronaut. Sci*. **7**, 189-198 (1940).
**16**	Steel	13,500	~10	*https://www.tibtech.com/conductivite.php?lang=en_US* R. L. Templin, et al. *J. Aeronaut. Sci*. **7**, 189-198 (1940).
**17**	S-silk composite	1,077	420	This work

*The toughness is estimated value from the data of current typical stretchable conductive materials or systems provided in the reference.

**The carbon fiber purchased from TORAYCA^{TM} .

Supplementary References

1. T. Lefevre, M. Pezolet, Unexpected beta-sheets and molecular orientation in flagelliform spider silk as revealed by Raman spectromicroscopy. *Soft Matter* **8**, 6350-6357 (2012).
