# Supplementary material for: A supertough electro-tendon based on spider silk composites
Source: Nat Commun. 2020 Mar 12;11:1332. doi: 10.1038/s41467-020-14988-5 (PMC7067870; doi:10.1038/s41467-020-14988-5)
Supplement: Supplementary file 3 — Description of Additional Supplementary Files [file 41467_2020_14988_MOESM3_ESM.pdf]

Descriptions of Additional Supplementary Files

Supplementary Movie 1
The spider we have raised for experiments. The spider, *Nephila pilipes,* was feeding on a locust in an 80 cm×60 cm×40 cm vivarium. The spider was kept at humidity above 65% and temperature about 25°. The spider was fed live locusts and flies three time a week. We collected the spider silk every two weeks using a scalpel for transfer within a rigid frame.

Supplementary Movie 2 and Supplementary Movie 3
The dynamic process of structural evolution of S-silk and S-silk@10% SWCNT under stretching. At the beginning of applying strain, the amorphous regions in natural S-silk gradually unfolded. In the case of the S-silk composite, the hydrophobic interaction between SWCNTs and silk proteins induced high strength. With increasing strain, the composite experiences higher normalized stress due to the bridge effect between SWCNTs and the silk protein. The S-silk fractured more easily than S-silk composite at a lower critical strain, showing the composite is much tougher. In the simulation, we defined fracture as the number of bridges crossing any cross section to be less than 1.

Supplementary Movie 4
The bending process of the humanoid robotic finger in response to different (low to high) touch forces. When we applied different pressures of 117, 327 and 749 Pa, the finger bent by 0°, 19°, 32° and 43°, respectively. Higher forces resulted in greater bending angles.

Supplementary Movie 5
We used the humanoid robotic hand assembled with the S-silk composite electro-tendon to grasp a green balloon. A tendon based on nylon, a common non-conductive material for robotic tendon, was used for comparison. The hand with the S-silk composite was able to catch the green balloon without deforming the shape of the balloon. This is because the pressure feedback system stopped the hand at a suitable pressure of 178 Pa. In the case of the robotic hand with the non-conductive nylon, the hand simply bent forward and failed to grasp the balloon because no signal was transmitted from the pressure feedback system to halt the hand.
